# Exploring How a Therapy Dog Intervention in a Tier 3 Classroom Influences Mental Health Components of Well-Being: A Case Study

**DOI:** 10.3390/bs15111585

**Published:** 2025-11-19

**Authors:** Kathleen M. Farrand, Jae Young Jung

**Affiliations:** Mary Lou Fulton College for Teaching and Learning Innovation, Arizona State University, Tempe, AZ 85287, USA; jjung57@asu.edu

**Keywords:** therapy dog, Tier 3, well-being, social emotional support, behavioral, animal-assisted interventions

## Abstract

The purpose of this case study is to examine how a therapy dog intervention can be integrated into a Tier 3 intervention classroom and how therapy dog interventions influence mental health components of well-being, social, emotional, and behavioral functioning, in a Tier 3 intervention classroom. This research used qualitative methods to triangulate data from classroom observations of the Tier 3 classroom with and without the therapy dog present including video data, field notes, student feedback, and a semi-structured interview with the classroom teacher/handler. Thematic analysis of transcripts from student feedback, semi-structured interview, and field notes was used for qualitative analysis. Multi-modal analysis was used to examine the phenomenon of the therapy dog intervention in the Tier 3 classroom and the multi-modal transcripts were aligned with the theme and sub-themes of the mental health components of well-being. The results indicated that a systematic integration model designed with a therapy dog intervention alongside a traditional Tier 3 approach can influence both emotional support and academic achievement. Therapy dog interventions positively impact the social, emotional, and behavioral well-being of students in Tier 3 settings when effectively integrated as a complementary intervention to enhance existing Tier 3 interventions.

## 1. Introduction

Educational systems worldwide face an escalating crisis in supporting students with behavioral and emotional challenges, requiring Tier 3 intensive interventions, which are the highest level of support for approximately 5% of students who have not responded to less intensive interventions (Wiedermann et al., 2023). Tier 3 interventions represent the most complex level of support within multi-tiered systems of support (MTSS) frameworks, characterized by highly individualized behavioral plans, specialized classroom environments, and comprehensive wraparound services (Walker et al., 2004). Students requiring Tier 3 supports typically present with chronic, severe behavioral challenges that significantly interfere with their learning and the learning of others, often accompanied by complex trauma histories, mental health concerns, and academic deficits (Scott et al., 2019). These students frequently exhibit patterns of escalating behaviors, including verbal and physical aggression, and disengagement in academic tasks, which necessitate intensive, multidisciplinary intervention approaches (Lane et al., 2019). The urgency of this challenge extends beyond individual student outcomes, affecting classroom stability, teacher retention, and the learning environment for all students (Sutherland et al., 2010).

Animal-assisted interventions (AAIs), particularly therapy dog programs, offer promising complementary support by providing non-judgmental social interaction (Beetz et al., 2012). School district-wide research identified three interconnected domains through which therapy dogs influence mental health components of well-being: social, emotional, and behavioral (Farrand & Jung, 2025b). While studies demonstrate positive AAIs outcomes, including improved reading skills, reduced anxiety, and enhanced social functioning (Brelsford et al., 2017), critical gaps remain. Specifically, little is known about how therapy dogs operate within intensive intervention contexts such as what distinct roles they play, how district-level support structures enable sustainability, and what systemic outcomes emerge from their integration. Addressing these questions is essential to move beyond documenting general benefits toward understanding how AAIs can be embedded within the most challenging educational environments.

Despite broad effectiveness evidence, not enough studies have systematically examined therapy dog implementation within Tier 3 intensive support settings where behavioral challenges are most acute and traditional interventions show diminished effectiveness (Cook et al., 2015). Education stakeholder analysis revealed that effects were prominent but least understood in these high-needs environments (Farrand & Jung, 2025a). This gap is problematic given that Tier 3 settings employ highly structured behavioral systems that may interact uniquely with AAIs (Scott et al., 2019).

### 1.1. AAIs in Education

AAIs have been increasingly adopted in educational settings to address diverse student needs, including academic engagement, socio-emotional development, and behavioral regulation (Beetz et al., 2012). Research indicates that therapy dogs provide non-judgmental companionship that fosters emotional safety and facilitates positive social interactions (Jalongo, 2015). AAIs have been associated with reductions in student stress, anxiety, and disruptive behaviors while simultaneously supporting improvements in self-esteem and classroom participation (Brelsford et al., 2017). Importantly, district-level studies demonstrate that therapy dog programs influence teaching and learning by enhancing relationships, promoting well-being, and strengthening school climate (Farrand & Jung, 2025a). These findings underscore the potential of AAIs as a complementary support mechanism in schools, though systematic evidence specific to high-intensity intervention contexts remains limited (Anderson & Olson, 2006).

### 1.2. Mental Health Components of Well-Being

Student well-being is commonly conceptualized across three interrelated domains: social, emotional, and behavioral functioning (U.S. Department of Education, Office of Special Education and Rehabilitative Services, 2021). The interconnection between social, emotional, and behavioral functioning creates a complex web of influences that determine educational outcomes for students with intensive needs (Zins et al., 2007). Student well-being is increasingly conceptualized across these three domains, which interact dynamically to influence learning, engagement, and long-term development (U.S. Department of Education, Office of Special Education and Rehabilitative Services, 2021). When interventions fail to address all three domains simultaneously, students are at risk of long-term disengagement from education (Maggin et al., 2017).

Social factors extend beyond basic interpersonal skills to include social problem-solving, perspective-taking, and the ability to navigate complex social hierarchies present in classroom environments (Gresham, 2016). Students with social skill deficits often experience failures across academic and behavioral domains, as peer rejection, social isolation, and misunderstanding of social cues compound existing challenges (Malecki & Elliott, 2002). In Tier 3 settings, where students are disproportionately vulnerable to peer rejection, targeted supports for social functioning are critical (Nitz et al., 2023). Research on AAIs indicates that therapy dogs can strengthen peer interaction, promote empathy, and foster a greater sense of belonging, thereby addressing social barriers to learning (Beetz et al., 2012).

Emotional factors encompass emotion recognition, regulation, flexibility, frustration tolerance, and recovery from emotional setbacks (Brackett et al., 2012). The neurobiological integration between emotional and cognitive processing means that emotional dysregulation directly interferes with working memory, attention, and executive functioning required for academic performance (Immordino-Yang & Damasio, 2007). Students in intensive intervention settings often exhibit emotional rigidity, delayed recovery times, and limited emotional vocabulary, impairing their ability to communicate needs effectively (Gueldner et al., 2020). AAIs studies demonstrate that therapy dogs reduce stress, facilitate emotional expression, and promote resilience, providing a complementary intervention to traditional emotional regulation strategies (O’Haire, 2013).

Behavioral factors involve the self-regulatory skills necessary to inhibit impulsive responses, maintain attention to task demands, and adapt behavior to changing expectations (Lane et al., 2019). Effective development of behavioral regulation requires supportive environments that emphasize self-monitoring and skill-building rather than relying solely on external compliance (Walker et al., 2004). Traditional Tier 3 interventions often overemphasize external control, which limits generalization and long-term maintenance of behavioral gains (Scott et al., 2019). By contrast, therapy dogs have been shown to increase motivation, enhance on-task behavior, and provide immediate nonverbal feedback, supporting students in internalizing behavioral regulation strategies (Hall et al., 2016).

The synergistic relationship among social, emotional, and behavioral domains means that intervention in one area can catalyze improvements across all three, while deficits in any single domain can undermine progress in others (Durlak et al., 2011). Recent district-wide analyses confirmed this interconnectedness, demonstrating that therapy dogs influence student well-being through overlapping pathways across domains (Farrand & Jung, 2025b). Theoretically, this aligns with ecological and resilience frameworks, which emphasize the importance of integrated supports that address multiple facets of student functioning within learning environments (Bronfenbrenner, 1979). This interconnected model suggests that the most effective interventions for students with complex needs are those that integrate social, emotional, and behavioral supports simultaneously, positioning AAIs as a uniquely comprehensive and relational approach (Fine, 2019).

### 1.3. Tiered Support Models and Intensive Intervention Contexts

Educational systems worldwide have adoptedMTSS as a comprehensive framework for addressing the diverse academic, behavioral, and social–emotional needs of students (Sugai & Horner, 2009). This three-tiered model provides increasingly intensive levels of intervention, with Tier 1 representing universal supports for all students, Tier 2 offering targeted interventions for students at risk, and Tier 3 delivering the most intensive, individualized supports for students who demonstrate persistent challenges despite receiving lower-tier interventions (Freeman et al., 2006). Within this framework, approximately 80–85% of students respond successfully to Tier 1 universal supports, 10–15% require Tier 2 targeted interventions, and 5% need Tier 3 intensive interventions (Sugai & Horner, 2020).

The implementation of Tier 3 supports occurs within specialized settings designed to provide individualized attention (Nitz et al., 2023). These environments typically feature reduced class sizes, increased staff-to-student ratios, and intensive behavior support plans that incorporate both proactive strategies and crisis intervention protocols (Gresham, 2016). However, traditional Tier 3 interventions often rely heavily on external behavior management systems, which may limit students’ development of intrinsic motivation and self-regulation skills necessary for long-term success (Cook et al., 2008). Research indicates that while these intensive interventions can produce short-term behavioral improvements, maintaining and generalizing gains across settings remains a persistent challenge (Maggin et al., 2017).

The sustainability of Tier 3 programs extends beyond student outcomes to encompass the well-being and retention of qualified staff who implement these complex interventions. Educators working in intensive intervention settings face unique stressors, including exposure to frequent aggressive behaviors, limited intervention effectiveness with the most challenging students, and emotional exhaustion from repeated crisis situations (Sutherland et al., 2010). High turnover rates among Tier 3 staff create additional challenges, as consistent implementation of individualized behavior plans requires sustained relationships and a deep understanding of each student’s specific needs and triggers (Pas et al., 2012).

Recent research has highlighted the need for innovative approaches that complement traditional Tier 3 interventions while addressing both student and staff well-being (Dreer & Gouasé, 2021). The integration of trauma-informed practices, restorative justice approaches, and strength-based interventions has shown promise in enhancing the effectiveness of intensive supports (Branson et al., 2017). However, implementation challenges persist, particularly in maintaining fidelity to evidence-based practices while adapting interventions to meet the complex, individualized needs of students requiring Tier 3 supports (Breitenstein et al., 2010).

Despite the growing body of research on MTSS implementation, significant gaps remain in understanding how complementary interventions, such as AAIs, can be effectively integrated within existing Tier 3 frameworks. The unique characteristics of Tier 3 environments create distinct implementation considerations that have not been systematically examined in the AAIs literature (Anderson & Olson, 2006). This gap is particularly concerning given that students in Tier 3 settings are among the most vulnerable in educational systems and may benefit from innovative, relationship-based interventions that address the underlying social, emotional, and behavioral factors contributing to their challenges.

The interconnected nature of academic, behavioral, and social–emotional functioning in Tier 3 populations underscores the need for comprehensive interventions that simultaneously address multiple domains of student functioning. Traditional approaches that focus primarily on behavior modification may fail to address the underlying emotional and social factors that contribute to persistent behavioral challenges, limiting their long-term effectiveness (Durlak et al., 2011). This creates an opportunity for complementary interventions, such as therapy dog programs, that naturally integrate support across social, emotional, and behavioral domains.

### 1.4. The Present Case Study

This case study aims to contribute to the growing body of qualitative research on AAIs by examining how a therapy dog influences a Tier 3 classroom, focusing on student outcomes and systematic integration. Building on prior district-level findings (Farrand & Jung, 2025b), this study conceptualizes therapy dogs not only as individual supports but also as integral components of intensive intervention systems. This dual focus examines how well-being domains intersect with the structural features of specialized education settings, positioning this case to contribute both practical insights for educators working in high-needs environments and theoretical implications for how AAIs can be conceptualized within MTSS. Guided by prior district level findings (Farrand & Jung, 2025a, 2025b) and gaps in existing AAIs literature, this case study was designed around the following research questions:

1. How do therapy dog interventions influence mental health components of well-being, social, emotional, and behavioral functioning, in a Tier 3 intervention classroom?
2. How can therapy dog interventions be effectively integrated within existing Tier 3 behavioral management systems to optimize student outcomes?

## 2. Materials and Methods

The study was conducted in accordance with the Declaration of Helsinki and approved by the Institutional Review Board of Arizona State University (protocol code STUDY00017481 and 11 April 2024), and it was also approved by the school district research and evaluation department. Informed consent was obtained from all subjects involved in the study.

### 2.1. Setting

The setting was a third and fourth grade combined classroom during the 2023–2024 academic school year in an elementary school located in a large school district in the southwestern United States. The elementary school has grades preschool through sixth grade and approximately 480 students. The school demographics consist of a minority student enrollment of 79% and 68% of the students are identified as economically disadvantaged.

The specific classroom for this research was designed as part of a Tier 3 intervention program so that students are provided with individualized and specialized behavior supports in a classroom setting with fewer students. The first through sixth grade classrooms in this program are designed not to have more than ten students and have one classroom teacher and one instructional assistant. For students to enter the program they must be referred by the school principal and the school must have tried all intervention and supports available, such as an evaluation for special education, informal and formal behavior plan, and academic interventions (when appropriate). If a student qualifies for special education services, then the Individualized Education Program team must determine if the child would benefit from placement in this classroom setting. The parent or guardian of the child must approve of placement in the program and specialized setting. The goal of the program is that each student will receive a 3-Tiered System of support that will allow each student to receive the support and interventions they need to be successful and return to their home school setting.

### 2.2. Therapy Dog Program

The classroom for this research was in a school district that has a district wide therapy dog program across 43 district locations. Therapy dogs in the program have gone through and passed specialized obedience and therapy dog training, as well as a final therapy dog evaluation before starting work as a therapy dog and handler team in the district. Therapy dogs attend work with their handler in their educational job, such as classroom teacher, principal, and occupational therapist, and participate in daily educational and office activities an average of one to three days each week during the academic year. The goal of the program is to support the social and emotional needs of students and staff throughout the school district.

### 2.3. Procedure

The first author emailed the classroom teacher/handler a recruitment email to participate in a semi-structured interview and classroom observations. Once the classroom teacher/handler identified that they wanted to participate in the study, the first author contacted the teacher/handler to identify a date that they could meet in person to go over the consent form, answer any questions they may have about the research, and drop off parent/guardian permission forms to be sent home with each student in the classroom. The classroom teacher/handler contacted the first author once all students had returned their signed parent/guardian permission forms to identify dates and a consistent time for the first author to visit the class and record classroom interactions of students during instruction with and without the classroom therapy dog. All participants (teacher, instructional aide, and students) had signed consent forms and students signed assent forms after the first author shared information about the research.

The first author came during scheduled math instruction time when the therapy dog was present and when the therapy dog was not present. The handler/teacher also participated in a semi-structured interview with the first author during noninstructional time. Upon completion of the semi-structured interview, the first author emailed the teacher/handler a link to a demographic survey to identify basic demographic information about the teacher and her therapy dog. Students in the classroom shared their opinions about the therapy dog before and after instruction during classroom observations by the first author. During classroom observations, video recordings were taken of instruction and interactions between teachers, students, and the therapy dog. The first author also completed detailed field notes after every observation to capture who was present, the nature of the classroom interactions, environmental information, and detailed information about interactions with and without the therapy dog present. In exchange for participation in the study, the classroom teacher/handler received a USD 50 Amazon gift card.

### 2.4. Participants

The classroom teacher/handler, referred to as Mrs. Gaddis [pseudonym], was a white female, in her forties, with a bachelor’s degree who had been teaching for eight years. She was also the handler for the therapy dog, Muffin [pseudonym]. Muffin was the pet of the handler and lived with the classroom teacher/handler. Muffin was a female rescue dog, and her breed was German Shepherd mix. The 2023–2024 school year was Muffin’s first year as a therapy dog in the district therapy dog program. Muffin attended school as a therapy dog approximately three days each week. On days Muffin was at school, she came to school with her handler prior to the start of the school day and stayed and worked alongside her handler the entire school day. The classroom had one instructional aide, referred to as Ms. Herrin [pseudonym], who was also present and actively involved in classroom instruction and interactions.

The student participants were a mix of third and fourth grade students. All five students (female = 1; male = 4) in the classroom for this research had a signed parent/guardian consent form and each student individually signed an assent form to participate in the research.

### 2.5. Data Collection

A case study approach was used to allow for an in-depth look at how the phenomenon of a therapy dog intervention in the context of a Tier 3 classroom. Specifically, an instrumental case study approach (Stake, 1995) was used to understand how a therapy dog intervention impacts the factors of mental health and well-being in a Tier 3 classroom. Case study allows for multiple data sources to be collected, as well as the viewpoints of different participants and their interactions to triangulate the data (Tellis, 1997).

#### 2.5.1. Semi-Structured Interview

The semi-structured interview questions were designed by the first author to learn more about the classroom teacher/handler’s experiences and motivations for being a handler of a therapy dog in addition to a classroom teacher. Also, the questions were designed to examine the classroom teacher/handler’s perceptions of the benefits of having a therapy dog in her classroom. The questions were included to learn more about specific examples of how the therapy dog had impacted specific students, teachers, and the school.

#### 2.5.2. Classroom Observations and Feedback

Classroom observations were arranged with the classroom teacher/handler during math instruction, each lasting approximately one hour, from 30 April 2024 to 9 May 2024. The first author came during scheduled math instruction time when the therapy dog was present, as well as days when the therapy dog was not present. Video recordings were taken by the first author of the students, teacher, instructional aide, and therapy dog interactions during math instruction using a handheld video camera. The observations took place in the classroom and sometimes extended to the adjacent field that was on the school campus. The classroom teacher/handler and instructional aide would often share their impressions of the therapy dog in the classroom before or after scheduled observation times. Students were also invited to share specific information about their impressions of having Muffin in the classroom before or after scheduled instruction when the first author was present.

#### 2.5.3. Field Notes

The first author took detailed field notes to describe the environment of the observation, who was present, information that was shared by participants, and specific interactions when the therapy dog was and was not present during and after each observation.

### 2.6. Data Analysis

The semi-structured interview with the classroom teacher/handler was transcribed by transcription software. Video recordings were transcribed by the first author of specific events, such as students sharing their feedback about having Muffin in their classroom and interactions between students and the therapy dog. Audio was not recorded and transcribed of students that were sharing personal information with the classroom teacher/handler and instructional aide when the researcher was present. The semi-structured interview transcripts, video recording transcripts, and field notes were anonymized to remove any identifiable information of the participants. Pseudonyms were assigned to all participants including the therapy dog.

Qualitative analysis was used to analyze the transcripts and field notes. Braun and Clarke’s (2006) thematic analysis method was used to analyze the transcripts through five steps that are described below. First, the authors read and reread all transcripts and field notes. Then, initial codes were created based off of previous research studies (Farrand & Jung, 2025a, 2025b) that analyzed a similar phenomenon of therapy dogs in educational settings. Next, data were grouped according to specific themes and themes were reviewed. In the final stage of coding, the themes and sub-themes were named and defined. The first and second author coded all data independently and then met to reach agreement on the naming and coding of themes and sub-themes.

Video recordings were recorded to inform multi modal analysis. The first author also made sure to check in daily with the classroom teacher/handler for member checking of interpretations of specific interactions in the classroom. Still image screenshots were taken of video recordings and a multi-modal transcription of what was observed was recorded to describe specific interactions during scheduled observations. The still images screenshots were made black and white to focus on the multimodal interactions in each image and red colored arrows and circles were added on top of the images to align with the multimodal description. Multimodal transcriptions were used in order to transcribe the interactions that were taking place to describe the physical environment, gestures, gazes, body position, tools, and proximity, as well as spoken language, when appropriate (Norris, 2004). Multimodal interaction analysis (Norris, 2004) was used to understand the multiple ways that the students, classroom teacher/handler, instructional aide, and therapy dog engaged. The multimodal transcripts were analyzed to align with the themes and sub-themes of the mental health components of well-being that were used for the interview transcripts, student feedback, and field notes in order to triangulate data about how the therapy dog intervention influences mental health components of well-being; social, emotional, and behavioral functioning in a Tier 3 intervention classroom. See Table 1 for the main themes and sub-themes, as well as the operational definitions of the sub-themes.

After coding was completed, the first author organized themes and sub-themes with representative illustrative quotes and multi-modal transcripts to describe how the therapy dog intervention influenced mental health components of well-being; social, emotional, and behavioral functioning in the Tier 3 classroom. Lastly, the classroom teacher/handler that participated in the study examined a rough draft of the writing to review the accuracy of the transcripts and interpretations of the data as a form of member checking (Stake, 1995) to inform final revisions of the analysis.

## 3. Results

In this section, we share examples of how the students, classroom teacher/handler, and therapy dog participated in classroom teaching and learning activities and how the therapy dog intervention influenced mental health components of well-being within three main themes: social well-being, behavioral well-being, and emotional well-being. The sub-themes are organized within the three main themes below in order of most identified to least identified. In addition, specific information about how the therapy dog intervention was integrated within the existing Tier 3 behavioral management system are described. All the examples included below come from the classroom teacher/handler interview, student feedback, field notes, and multimodal transcripts of classroom observations, but due to space limitations, we do not include the full transcripts or multimodal examples for each sub-theme. Pseudonyms are used for all participants, including the therapy dog, and all adult participants had signed consent forms, and all student participants had signed consent forms from a parent/legal guardian for their image to be included without blurring.

### 3.1. Social Well-Being

The theme of social well-being was identified the most and had six sub-themes: environment, peer relationships, supporting specific students, community, belongingness, and empathy/compassion.

#### 3.1.1. Environment

**Figure 1:**
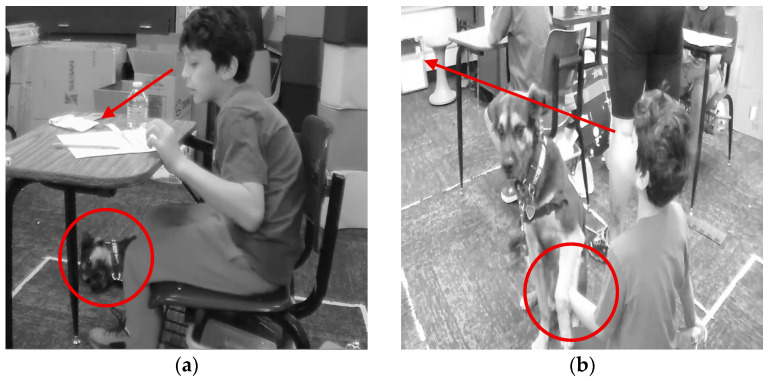
This is a figure of Cade during instruction with the therapy dog in the classroom environment. Notice the white tape on the carpet around him in both figures indicating space that he can move around within the classroom during activities: (**a**) Cade is sitting at his desk in his white square on the carpet as he pets Muffin with his right hand. A red circle was added around Muffin to show her proximity to Cade during this interaction. Cade is looking at his math work on his desk and working on answering math questions with his left hand on the paper on his desk. A red arrow was added from Cade pointing downwards towards his math work to visualize his gaze. (**b**) Cade is sitting on the floor with one arm reaching out towards Muffin as she lays a paw on his arm. A circle is added around Cade’s arm with Muffin’s paw to visualize this interaction in the figure. His gaze is focused on the math instruction in the front of the room with a red arrow pointing from Cade to the front board to visualize his gaze towards the math instruction.

The sub-theme of environment was identified the most within all sub-themes. Environment was identified for instances that described the therapy dog having a positive impact on the educational environment. The desks in the classroom are surrounded by white tape on the floor that indicate space where each student can move around within during instruction. Below in Figure 1, a student is shown engaging at his desk and then on the floor with Muffin during math instruction. In both instances he is actively working on his math work within his white box on the floor and moving from his desk to the floor, while still petting and engaging with Muffin with one of his hands. Muffin positively motivated Cade to move within his learning environment and actively participate in math instruction.

#### 3.1.2. Peer Relationships

Peer relationship as a sub-theme refers to instances when the students, teacher, or instructional aide had an interaction or social connection because of the therapy dog. According to Mrs. Gaddis:

*When you realize that these kids don’t always necessarily build each other up…it’s really awesome when they do that. And lately…they are starting to help each other and, hey, I got this. This is why. And I kind of let that go because that’s the relationships they need to learn how to build. And honestly, the Danielson model that we’re evaluated with encourages that as well, is building the kids up to a point where they can take it and run and help each other and not always get input from us.*

An example from a classroom observation that was categorized as peer relationships was during math instruction when students were being asked to identify true and false statements about math concepts. The students, teacher, and instructional aide are interacting and encouraging each other in a positive way during math instruction while Muffin walks around them and takes turns walking by and sitting with students for pets. Ms. Herrin, the instructional aide, was using the SmartBoard in front of the class and asking the class questions, while Mrs. Gaddis, the classroom teacher/handler, was walking around the room to check in one-on-one with students to assess understanding and provide more personalized support. Muffin continues walking around to the students, and they pet her as she comes by them. The following is part of the dialogue describing an interaction between two students as Muffin was walking around them in the classroom. The students are interacting in a positive way during math instruction in part due to the presence of Muffin and the close proximity and comfort she provides each student which contributes to their social connection.

Ms. Herrin: *Is it a true statement?*

Triston: *Yes*

Cade: *Woo! Let’s go!*

Ms. Herrin: [Cheers loudly and dances to celebrate as she hands Triston a positive ticket.]

**Figure 2:**
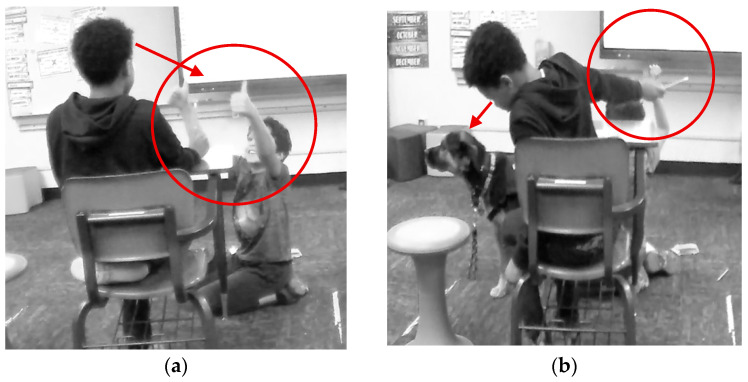
This is a figure of Triston and Cade participating and encouraging each other during math instruction with the therapy dog present: (**a**) Triston is sitting up straight in his chair gazing at Cade after he answers his question with a red arrow pointing from Triston towards Cade to visualize his gaze. Cade is giving him two thumbs up and smiling as he gazes back at Triston. A red circle is included around Cade smiling with his two thumbs raised in the air to draw attention to his use of hands and facial features to encourage Triston. (**b**) Triston is gazing at Muffin with his right arm outstretched to raise it to answer a question. A red arrow was added from Triston downwards towards Muffin to visualize his gaze. Cade is sitting on the floor in front of Tristan with his left arm petting Muffin and his right arm raised to answer the question. A red circle has been added around Triston’s raised arm and Cade’s raised hand to draw attention to their visual cue of their participation in the math instruction.

Figure 2 shows the above situation where Triston is sitting up proud as he answers a question correctly that the instructional aide asked. Cade is smiling, has two thumbs up, and then goes on to cheer for Triston after he answers the question correctly. Cade and Triston are demonstrating a social connection, encouragement, and active participation in the math activity with Muffin present and in close proximity.

#### 3.1.3. Supporting Specific Students

The supporting specific students sub-theme was identified in instances where someone indicated that the therapy dog intervention was connected to individualized support for an individual student. It was also associated with examples where a Tier 3 intervention was identified for supporting a specific student. In the example below, a student shares how Muffin [therapy dog] knows when a student is sad and provides individualized support. According to Logan:

*Sometimes when I get sad and go off to the corner….She [Muffin] kind of knows when you are sad. She’ll come up to you and lay right next to you. And then you pet her and it’s kind of nice because she kind of knows.*

**Figure 3:**
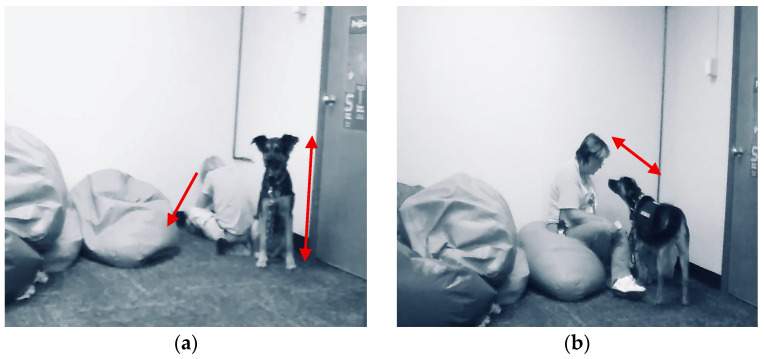
This is a figure of Logan during instruction with the therapy dog: (**a**) Logan is sitting in a corner of the room looking down at the floor with a red arrow pointing from Logan towards the floor to visualize his gaze. Muffin is sitting directly behind him with her ears raised and her gaze looking out for the classroom teacher. A red arrow has been added pointing up and down on the right side of Muffin to emphasize her sitting up with a tall posture. (**b**) The classroom teacher is sitting on a bean bag chair next to Logan and looking at Muffin who is now standing with her ears relaxed and looking at the classroom teacher. A red arrow is added pointing from Mrs. Gaddis to Muffin to visualize the two of them looking at each other.

The above quote was also connected with a multimodal observation of Logan removing himself from instruction and going off to the corner. Figure 3a shows Logan sitting in the corner with his head and gaze downwards towards the floor away from the other people and instruction. Muffin comes over shortly after Logan sits down and sits up straight directly behind him with her ears raised and her gaze looking towards Mrs. Gaddis as if to indicate that Logan needs support. Next, shown in Figure 3b, Mrs. Gaddis has come and sat down on the bean bag chair next to Logan and is looking at Muffin as if to confirm that she knows that Logan needs specific support.

**Figure 4:**
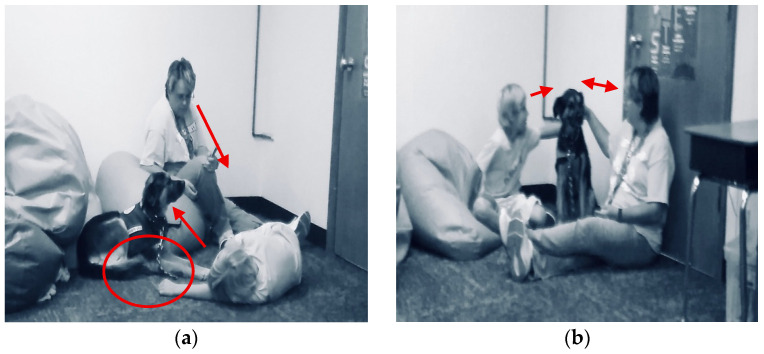
This is a continuation of Figure 3 of Logan with the therapy dog and his teacher: (**a**) This figure shows Logan laying on the floor with his gaze and arm outstretched towards Muffin with a red circle added around Logan’s arm and Muffin to draw attention to his outstretched arm. A red arrow is shown pointing from Logan towards Muffin to visualize his gaze. His teacher is sitting next to them on the bean bag gazing at Logan. A red arrow has been added to visualize her gaze towards Logan. (**b**) Logan is now sitting on the bean bag smiling with his arm on Muffin petting her. His teacher is sitting on the ground next to them gazing and petting Muffin while they talk. Arrows have been added from Logan and Mrs. Gaddis towards Muffin to visualize both of their gazes towards Muffin.

Mrs. Gaddis and Logan begin to talk. In Figure 4a, Logan has now moved out of the corner and is laying on the floor next to Muffin and Mrs. Gaddis. Logan’s arms are outstretched petting Muffin, and his gaze has also shifted towards Muffin. Mrs. Gaddis is now looking at Logan as they talk. Then, as shown in Figure 4b, Logan moves up to the bean bag chair and Mrs. Gaddis moves to sitting on the floor, so both of them can look at and pet Muffin as they talk. Logan is now smiling as he pets Muffin and shortly after rejoins math instruction. In multimodal Figure 3 and Figure 4, as well as Logan’s comment reflecting on this and similar scenarios, Muffin and Mrs. Gaddis are supporting a specific student. Muffin notices a student needs support, she moves in close proximity to the student, alerts Mrs. Gaddis, and stays with the student until he is ready to return to the class activity. Mrs. Gaddis notices that a student is in the corner and has had some time with Muffin. She joins the student on the floor and provides individual support to the student through conversation, proximity, and listens and talks with him until he has identified why he was upset and how he can rejoin the group and participate.

Mrs. Gaddis reflected on the above situation where Muffin provided personalized support for a student. Mrs. Gaddis stated:

*She [Muffin, therapy dog] helps calm him just by sitting there and she sits and waits. And then the second that he starts engaging, then she becomes more engaged in the situation. And I didn’t ever teach her that, but she seems to understand what the kids need and it’s really kind of cool to watch. And with Logan, that’s why I came back too, and I sat with him for a while. I was like, “Hey, I really need help with these answers. If you could give me the answer, then I can tell if you are getting it for sure or not?” And so, since he could sit back there with both of us and he could give answers, but it wasn’t disrupting the rest of the class, it works for him. So, with him, I have to approach him on several different levels to get him to come down, but she helps quite a bit just because of her calming, lying next to him. She’ll put her foot on him, “Hey, pet my tummy, or whatever.”*

The above statement demonstrates that Muffin and Mrs. Gaddis provide personalized support based on their knowledge of the individual needs of each student.

#### 3.1.4. Community

Community was associated with comments and instances where Muffin was described as being part of the group within the classroom or school. Mrs. Gaddis explained that Muffin has become a part of the classroom and larger school community at her school. According to Mrs. Gaddis:

*She [Muffin, therapy dog] goes to every staff meeting with us. And I think one day I didn’t take her, and I had to come back to my room to get her because there were a lot of very disappointed people. So, I think she’s just as much a part of the staff and what’s going on here as she is with the students.*

Muffin is also a part of the community that brings together students across the Tier 3 classrooms at her school. Mrs. Gaddis described how Muffin supports and motivates students with making good choices with behavior and their academic work and contributes to the larger Tier 3 community by going on a walk or having a short visit with a student. Mrs. Gaddis shared:

*We have all 1, 2, 3, 4 classrooms all in a row, generally what we do is leave the two doors open and she’ll [Muffin, therapy dog] go between the two to four classrooms and greet the kids. And actually today, our fifth and sixth grade classroom, the kids came over at the end of the day, they were testing and they were like, “Muffin didn’t come in today.” And I was like, “Yeah, well the door was shut because you guys are testing.” And then, the rest of the kids, kindergarten and then first, second grades next to us as well. Sometimes she’s earned like, “Hey, I need a break. Can I please go out? Can I take Muffin?” And that motivates a couple of the kids.*

The students at Muffin’s school look forward to her consistent presence in their classroom and school.

#### 3.1.5. Belongingness

Belongingness refers to instances that demonstrate a sense of membership. Mrs. Gaddis and Ms. Herrin reflected on how the presence of Muffin in the classroom has supported them and indicated that Muffin knows the kids and is able to support students, like the staff do, as they regulate their behavior.

*Helps me immensely, because she [Muffin, therapy dog] knows our kids. She knows and has the ability to comfort our kids. If one of our kids is dysregulated, she knows before we do sometimes. And she picks up on it really easily. And it’s such a comfort that they can just calm down and hang out with her. And she helps regulate the kid back to what they are supposed to be doing. As staff, it is significantly helpful.*

Muffin is a member of the class and serves like a co-educator in her ability to sense when a student needs support and provide that comfort to the student.

#### 3.1.6. Empathy/Compassion

Empathy/compassion was identified as a sub-theme for comments and instances that reflected individuals supporting each other with a sense of empathy and compassion. Muffin has supported the students in her class with extending social emotional learning (SEL) concepts that are being introduced in a larger group setting to their classroom. Students have begun to make connections between what they are learning about feelings and emotions and start making connections with Muffin to better understand SEL concepts, like empathy and compassion. According to Mrs. Gaddis:

*We incorporate that [social emotional learning] in our learning every single day because our kids, especially with having behavior disorders, they’re told all the time, “You are bad. You are this, you are that.” And so, they get a lot of that negative input. So, when they come into this program, we do social emotional learning five days a week, every morning we do it. And it’s funny because Muffin [therapy dog] now joins our circle and the kids will include her like, “Oh, well if we did this, she wouldn’t like that.” It’s been good because animals’ emotions are very much on their sleeve because they’re not going to hide it. They don’t know that they should or shouldn’t. So, I think that that does help the kids a little bit to learn forgiveness, how to react.*

In addition, Mrs. Gaddis described how having Muffin in the classroom has supported empathy and compassion by understanding that bad feelings happen and calming down from those feelings is okay too. She explained:

*I think that seeing is believing, but also just to understand how much it affects the kids and how much they look forward to the interaction with the dog and how quickly it does deescalate situations that are going in a wrong direction. I think being an animal person to begin with, I just think that’s so important for kids to learn and to understand that it’s okay. It’s okay to have bad feelings, and it’s okay to calm down from those feelings.*

### 3.2. Behavioral Well-Being

The theme of behavioral well-being was the theme identified the second most and had five sub-themes: academic engagement, self-regulation, motivation, responsibility, and job satisfaction.

#### 3.2.1. Academic Engagement

The sub-theme of academic engagement was identified the second most amongst all sub-themes. Academic engagement was categorized for comments and instances that referred to how someone was involved in an academic task. Logan shared that Muffin helps him when he needs support during learning activities.

*Like when we are working and we don’t really know the answer, she [therapy dog] will walk up to us and we can pet her. I pet her and it helps reset my mind and I can figure out the answers and stuff…It has been working for me, and it can help us.*

**Figure 5:**
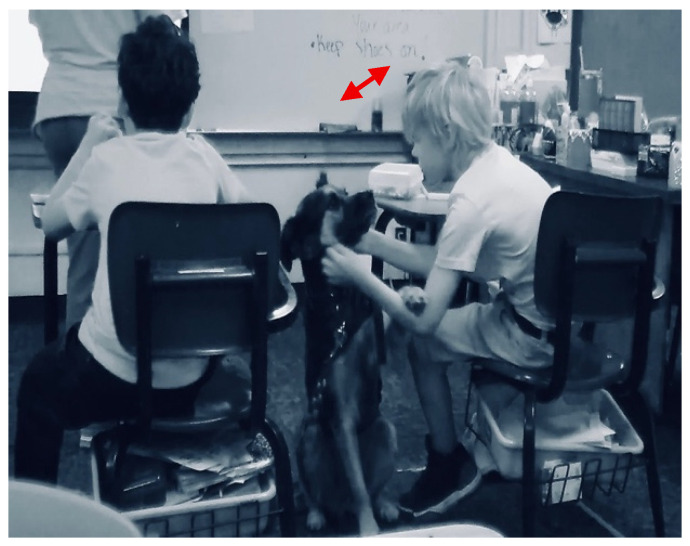
This is a figure of Logan sitting at his desk with both of his hands on Muffin’s face petting her. Logan and Muffing are gazing at each other, and Muffin has her paw on his arm. A red arrow pointing from Logan to Muffin has been added to visualize the two looking at each other.

Figure 5 shows Logan taking a brief break during math instruction to pet Muffin before returning to the whole group math activity. Logan gently pets Muffin with both hands, and they gaze at each other for a bit and Logan refocuses on his math work.

#### 3.2.2. Self-Regulation

Self-regulation was identified as a sub-theme in instances and quotes that indicated that the therapy dog supported someone with dysregulated behavior. Mrs. Gaddis reflected on students with dysregulated behavior before Muffin and now that Muffin is in the classroom. She shared:

*Prior to having her [Muffin, therapy dog], I feel like there was a lot more negotiating with the kids on trying to get behaviors to come down. And sometimes it would take me 45 minute, an hour, all day depending on how far they escalated. But I feel the difference with having Muffin here is we can de-escalate it a lot quicker. It doesn’t go as high. It doesn’t mean that all behaviors are gone because we still have behaviors. But I think between the structure that I have in my classroom and then having her as a tool, it helps us de-escalate a lot quicker with the kids and give them options that they like and that they can calm down and not feel like anyone’s judging them.*

The presence of Muffin combined with the structure and strategies already in place in the classroom have led to students regulating their emotions quicker and having a non-judgmental confidant that they can talk to when working on regulating their emotions.

**Figure 6:**
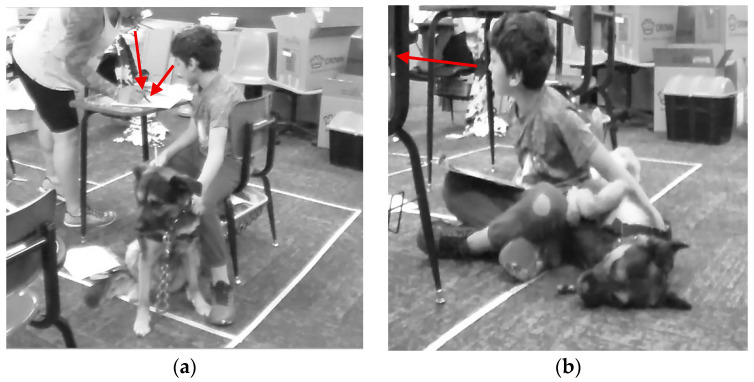
This is a figure of Cade working in his learning environment after Muffin has supported him with regulating his emotions: (**a**) Cade is sitting at his desk in his white square on the carpet as he pets Muffin. Cade and Ms. Herrin are both looking at his math work on his desk with two red arrows shown pointing downwards to visualize their gaze towards the math work. (**b**) Cade is sitting on the floor with one arm petting Muffin. His gaze is looking at the teacher in the front of the room, and a red arrow has been added pointing from Cade to the front of the room to visualize his gaze. Cade’s math activity paper is on a clipboard on his lap that he is using to figure out the answers to math problems.

An example from the field notes and multimodal transcripts identified how Muffin supported a student with regulating their behavior. Two students were playing with a fidget item on the floor, and they were asked to go back to their seats and no longer play with the broken item. Cade was visibly frustrated as he went back to his desk. Muffin came over to Cade almost immediately after he returned to his desk and his mood improved (see Figure 6). Cade began petting Muffin with one hand and working on his math work with the other hand at his desk. Cade actively participated in learning and Muffin supported him with staying engaged in his learning environment as he petted her. Cade stayed within his learning environment in the classroom that was sectioned off with white tape on the floor. Cade was able to transition from working at his desk to working on the carpet with a clipboard as he continued to pet Muffin and actively work on his math work and participate in the whole group activity.

Mrs. Gaddis shared an example of how Muffin supported a student in the classroom earlier in the school year with regulating their behavior.

*When he [student] gets upset, he kicks things, and he’ll hit himself or scratch himself…Muffin will just walk over and sit next to him. And one day, he was kicking his leg on his chair, and I was sitting here watching it because I was in a little bit of disbelief. She walked over, sat next to him for a minute, he kept kicking the chair. She actually put her head down where he was kicking, and I thought, “Okay, this is not, I don’t know about this.” And he’s very animal oriented himself. He’s one of the ones that was really looking forward to the dog. He stopped kicking, all of a sudden, his hand came down, he started petting her. It calmed the situation in less than a minute.*

#### 3.2.3. Motivation

Motivation as a sub-theme aligned with quotes and instances where the presence of a therapy dog in an educational space motivated someone to do something. Mrs. Gaddis described how Muffin can be a motivating presence for the students in her classroom.

*They’re [students] always like, “Can she [Muffin, therapy dog] sit next to me? Can she sit?” And I’m like, “Guys, first off, there’s only five of us, so she can sit next to all of us.” But it’s kind of, yeah, they want to make sure that they all have a little piece of her, of time with her, and that does motivate them to learn. As long as you’re weaving that piece into your classroom.*

The above quote aligns with the presence of Muffin having a positive impact on the classroom environment and motivating students to want to learn and spend time with her.

**Figure 7:**
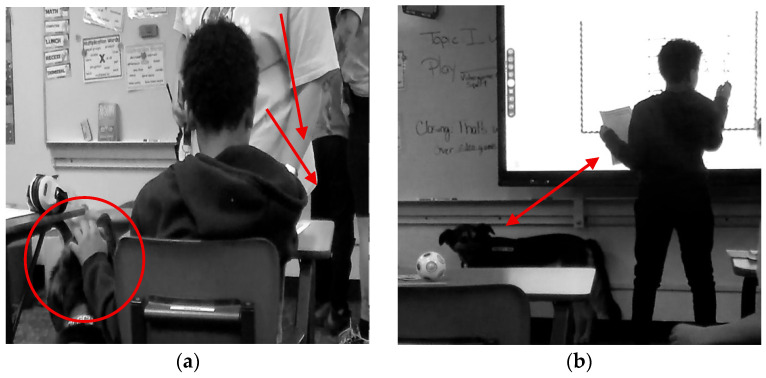
This is a figure of Triston participating during math instruction with Muffin by his side: (**a**) Triston is sitting at his desk with his left-hand petting Muffin. A red circle is around Triston’s hand petting Muffin. Triston’s right hand is holding a fidget ball, and his gaze is downwards toward his math work with a red arrow pointing downwards to visualize his gaze. Mrs. Gaddis is standing in front of him and looking and pointing at his completed math work as they talk. A red arrow is also added to visualize Mrs. Gaddis’s downward gaze. (**b**) Triston then goes up to the front of the room to write his work on the board to share with the class. He is holding his math work in one hand and writing on the board with the other hand. Muffin stands next to Triston as he writes his math work on the board. His fidget ball is sitting on top of his desk. A red arrow pointing at Muffin and Triston is included to visualize their close proximity to each other.

On a number of occasions, Muffin’s presence in the learning space motivated students to complete their math work and actively participate in whole group and one-on-one instruction. In this instance, Triston is petting Muffin as he completes his math work on fractions at his desk and then Muffin walks up with him when it is his turn to write and share his work on the board. He is engaged and responsive when asked questions one-on-one by Mrs. Gaddis and he shares his work with the whole group when it is his turn. See Figure 7 for the multimodal description of how Muffin motivated Triston during math instruction.

Teacher: *How are you doing Triston?*

Triston: *Ok*

Mrs. Gaddis: [She looks and points at his math work on his desk]. *This is right. Both of those are right. When Logan goes and sits down, can you write those two things on the board?*

Triston: [Shakes his head yes]

Mrs. Gaddis: *Can you do that?*

Triston: [Shakes his head yes]

#### 3.2.4. Responsibility

Responsibility as a sub-theme was associated with quotes and instances where the presence of a therapy dog supported a feeling of responsibility. According to Mrs. Gaddis:

*What I’ve noticed is the kids, before when they would do those social emotional questionnaires, they would just click, click, click, click, click. Now, they actually read it. So, they actually, sometimes they’ll ask me, “What does this mean?” And then, I have to explain it to them, but I feel like they’re actually taking the time to think about it more than they did in the past, which is interesting to me because you wouldn’t think that a dog would make any bit of difference there.*

The above quote demonstrates that students are being more responsible in how they engage with describing their social and emotional behaviors and how they complete tasks in the classroom due to the presence of Muffin.

#### 3.2.5. Job Satisfaction

Job satisfaction was aligned with quotes and instances where the presence of a therapy dog has improved one’s belief in their work and educational job. Mrs. Gaddis reflected on having Muffin with her in the classroom this academic year as a therapy dog and how it has brought her additional joy in her job. Mrs. Gaddis shared:

*It benefits me in a lot more ways than I ever thought. For one, it helps me calm outbursts a little bit quicker because you can, “Hey, do you want to go for a walk? Do you want to take Muffin [therapy dog]?” You can’t kind of get that disengagement because you can bring them down…I mean, she helps all over the place. I can’t even think of everything that she does. But I noticed the kids, even the kids that don’t know her yet that are coming into our program next year from another site, they’re already like, “Hey, can we get a picture of Muffin?”*

Mrs. Gaddis explained how Muffin has supported students with engagement, motivation, and regulating emotions. In addition, she described how good Muffin is at her job and how grateful she is that they are able to work together as a handler and therapy dog team.

*Yep. She’s [Muffin, therapy dog] really good at it. Like I said, she was meant to do this job and for some reason she came to me and thank God I’ve been able to do that with her because she’s really good at what she does.*

### 3.3. Emotional Well-Being

The theme of emotional well-being was identified the least of the three themes and had four sub-themes: joy/happiness, stress relief, self-efficacy, and non-judgmental/trauma informed care.

#### 3.3.1. Joy/Happiness

**Figure 8:**
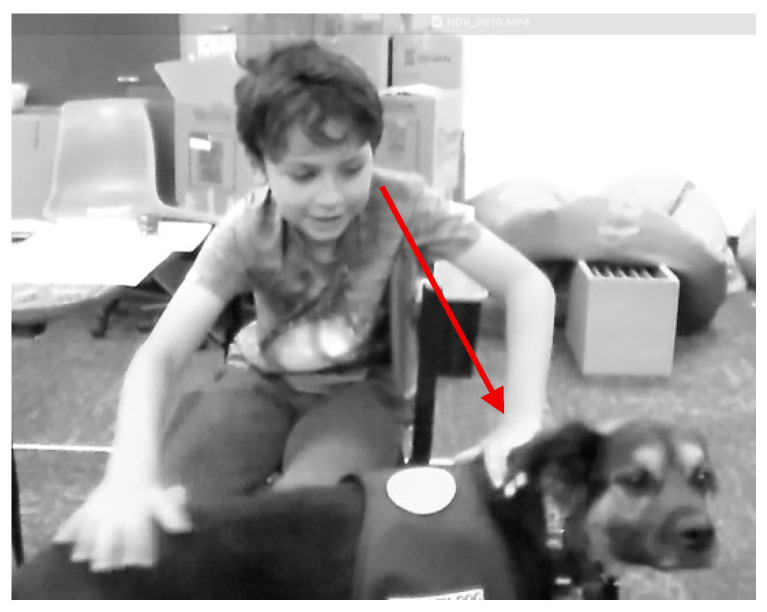
This is a figure of Cade smiling and gazing at Muffin with a red arrow added pointing from Cade to Muffin to visualize his gaze. He is petting Muffin with both hands as he takes a brief break from completing his math work. Muffin stands with her ears relaxed next to him.

Joy and happiness were combined as sub-theme for quotes and instances that reflected a sense of positive feelings or visible examples of joy, such as smiling, due to the presence of the therapy dog at school. Figure 8 is an example of joy and happiness in the classroom. Cade is smiling as he takes a brief happiness break to pet Muffin before he returns to his math work at his desk.

Mrs. Gaddis reflected on how much Muffin enjoys being a therapy dog. Mrs. Gaddis shared:

*I should probably just do a video of the morning when she knows it’s her morning. She gets so excited about coming to school. It’s a high-pitched yappy yapperton and she runs around the yard like a maniac. I mean, it kind of takes us a few minutes to get to the car because she’s so overexcited about it. She also, once we’re in the classroom, I’ve never taught her how to read time, but apparently, she knows it because she knows when it’s time to go up to get the kids off the bus. She is on me like, “Let’s go, let’s go, let’s go.” So, she very much likes what she does, and she tries, like yesterday in the morning she tried everything to get to go to school with me and I was like, “It is not your day, ma’am…You just have that feeling that she knows what her job is.*

#### 3.3.2. Stress Relief

Stress relief was identified as a sub-theme for instances and quotes that indicated that the presence of the therapy dog made someone relieve stress or feel better. A student shared, “If we’re sad. She [Muffin, therapy dog] like knows when we are sad. She walks up to us and she likes to lay down next to us. And that’s kind of nice because we can lay with her.” The student describes how spending time with Muffin supports them with feeling better and that Muffin even seems to know when a student needs her presence.

#### 3.3.3. Self-Efficacy

The sub-theme of self-efficacy was associated with quotes and instances where the presence of a therapy dog made someone believe in their own abilities to do something. The presence of Muffin in the classroom to sit with students and provide them with individualized support often led to Mrs. Gaddis and Ms. Herrin having more time to go around the classroom for individual check-ins with each student to assess understanding of content and ask about their day. Muffin will often sit with students as they are working individually to provide additional support and belief that they can do it, while Mrs. Gaddis and Ms. Herrin provide specific positive praise and encouragement to support each student’s belief in themself.

According to Mrs. Gaddis:

*He [student] just lacks confidence sometimes. And yesterday it was like, hey, I know you know this. So, then we were just kind of, I tell him, “This is our secret. It’s only between us.” And so then, he’ll tell me, “Okay, I think that’s this amount of money.” And I’m like, “Yeah, that’s great.” He was, because sometimes that’s just all they need. They don’t want to call out in front of everyone, but they do know the topic, they just worry that they don’t…She’ll [therapy dog] sit with him a lot as well during those kinds of things.*

See Figure 2 under peer relationships for an example of students encouraging each other and contributing to self-efficacy in their peers with the presence of Muffin for a classroom example of self-efficacy.

#### 3.3.4. Non-Judgmental/Trauma Informed Care

The sub-theme of non-judgmental/trauma informed care was aligned with comments and examples where the therapy dog intervention was used to support how individuals felt or acted. Mrs. Gaddis shared that she, Ms. Herrin, and Muffin all look at each student as an individual and provide individualized strategies to support how they feel and act. The classroom teacher/handler shared:

*He’s my go-for-a-walk kid. He’s always wanting, he usually likes to remove himself from the situation, calm down and then come back. So yeah, we look at all of them as individuals. And when Muffin [therapy dog] is not here, they all have different things that they like to do.*

**Figure 9:**
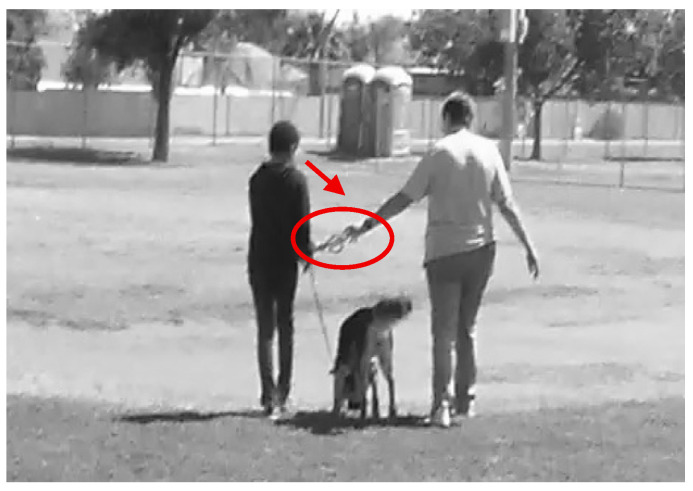
This is a figure of Triston walking outside with Muffin and Mrs. Gaddis. Triston and his teacher are both shown holding the leash, a red circle is around their hands on the leash, as Muffin walks in between them. Triston’s gaze is towards Muffin and his teacher with a red arrow pointing downwards from Triston to visualize his gaze.

Triston reflected on what he liked about Muffin and shared, “Going on walks.” Both of these quotes aligned with Triston getting support from Muffin, as well as his teacher and instructional aide through walks. Triston could ask or Mrs. Gaddis or Ms. Herrin may inquire to see if Triston would like to go for a walk to think and talk about what is on his mind, identify a solution if there is a problem or concern, and return to the classroom for teaching and learning activities. For example, Triston was not engaging during math instruction, so Mrs. Gaddis invited him to go for a walk with her and Muffin. While walking with Muffin and Mrs. Gaddis, Triston shared a problem he was having, he came up with a solution and then returned to the classroom and actively participated in math instruction. Figure 9 shows Triston holding the leash with Mrs. Gaddis as they walk with Muffin. Triston is able to talk in a non-judgmental space with Muffin and Mrs. Gaddis about how he is feeling and come up with a solution, so he feels supported and ready to learn.

## 4. Discussion

This investigation provides the first systematic examination of how therapy dog interventions can be effectively integrated within Tier 3 intensive support systems while influencing multiple domains of student well-being. Through intensive qualitative methodology using a case study approach, this research demonstrates that AAIs function as complementary enhancements rather than replacements for existing evidence-based practices, creating synergistic effects that address the complex needs of students requiring the highest level of educational support.

**Figure 10:**
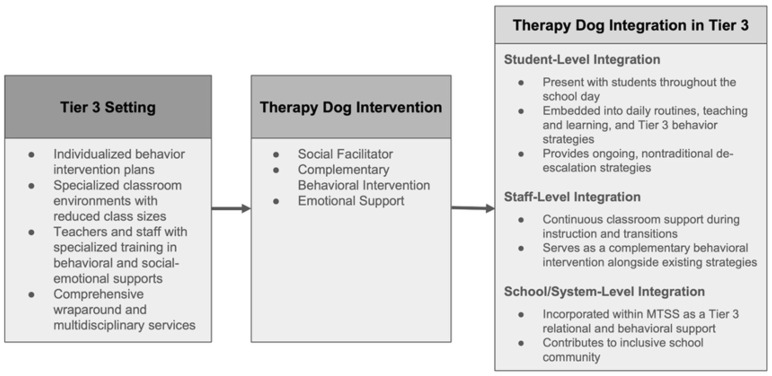
This figure describes how a therapy dog intervention can be effectively integrated within existing Tier 3 behavioral management systems. MTSS = multi-tiered systems of support.

The systematic integration model illustrated in Figure 10 offers insights into how therapy dogs can work alongside traditional Tier 3 approaches. These findings suggest that, rather than requiring separate programming or competing with existing evidence-based practices, therapy dogs may be embedded within established Tier 3 systems as complementary enhancement tools. This finding extends prior implementation perspectives that often position innovative interventions as alternatives to existing practices (Cook et al., 2015), by demonstrating instead that therapy dogs can function as amplifiers of established Tier 3 interventions. This complementary approach aligns with research emphasizing the importance of multi-faceted support systems for students with complex needs (Durlak et al., 2011; Scott et al., 2019).

The Tier 3 setting components depicted in Figure 10 reveal why intensive intervention contexts may be uniquely suited for AAIs implementation. The combination of individualized behavior intervention plans, specialized classroom environments with reduced class sizes, teachers with specialized training, and comprehensive wraparound services (Gresham, 2016) creates optimal conditions for therapy dog integration that may not exist in general education settings. These findings suggest that the effectiveness of AAIs may depend critically on matching AAIs implementation strategies to environmental characteristics and student population needs.

The therapy dog roles illustrated in Figure 10 represent an expanded conceptualization beyond traditional perspectives that emphasize individual comfort provision. The social facilitator function demonstrates how peer relationships can be supported in Tier 3 settings, where students typically have histories of social rejection and negative peer interactions as documented in prior research (Malecki & Elliott, 2002). The complementary behavioral intervention role indicates that therapy dogs can be integrated with existing behavioral support systems rather than operating separately from them. This integration approach is consistent with recommendations for multi-component interventions in intensive settings (Lane et al., 2019; Walker et al., 2004). The emotional support function operates differently in Tier 3 contexts than in general education settings, providing trauma-informed care that addresses the complex emotional needs characteristic of students requiring intensive intervention.

The three-level integration structure demonstrates the sophisticated coordination required for effective AAIs implementation while revealing why superficial or poorly integrated programs may show limited effectiveness. The systematic embedding across daily routines, instructional activities, and existing behavioral strategies suggests that comprehensive integration across multiple levels may be more effective than treating therapy dog interactions as discrete interventions. This finding aligns with research on implementation science emphasizing the importance of systematic integration for intervention success (Breitenstein et al., 2010; Freeman et al., 2006). This multi-level framework suggests that AAIs effectiveness may depend more on systematic integration quality than on specific intervention techniques or therapy dog characteristics.

**Figure 11:**
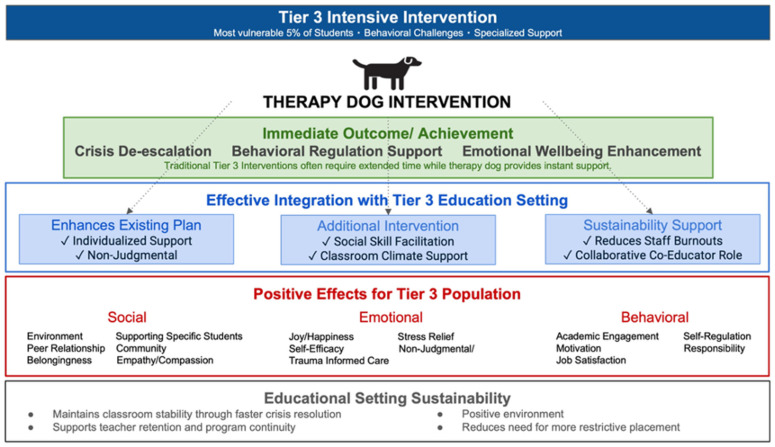
This figure describes how a therapy dog intervention can be effectively integrated within existing Tier 3 behavioral management systems.

The model presented in Figure 11 demonstrates how therapy dogs can create systemic transformation through interconnected domain effects, extending individualistic conceptualizations of AAI mechanisms. These findings indicate that the dominance of social well-being outcomes over emotional and behavioral effects may reflect the relational nature of therapy dog interventions, offering an alternative perspective to prevailing theoretical frameworks emphasizing individual stress reduction and comfort provision (Beetz et al., 2012; O’Haire, 2013).

The social well-being effects illustrated in Figure 11 suggest that therapy dogs may function as environmental modifiers in addition to serving as individual therapeutic providers. This dual function provides evidence that supports examining classroom conditions as modifiable intervention targets rather than fixed constraints, building on research demonstrating the malleability of educational environments (Lane et al., 2019; Sugai & Horner, 2009). The evidence that therapy dogs can systematically reshape social dynamics through environment, peer relationships, and community building suggests that AAIs may address fundamental contextual factors that contribute to behavioral difficulties in ways that complement traditional interventions focused on individual skill deficits (Gresham, 2016; Walker et al., 2004).

The environmental transformation documented in this research operates through mechanisms that traditional behavioral interventions cannot access. While traditional approaches rely on structured activities and explicit instruction to build social skills, the current findings suggest that therapy dogs create natural collaboration opportunities and reduce social inhibition through their mere presence, potentially enhancing the effectiveness of structured social skills instruction. This complementary relationship between AAIs and traditional approaches warrants further investigation across diverse Tier 3 settings. The peer relationship facilitation effects indicate possibilities for relationship repair in Tier 3 settings, where students typically have extensive histories of negative peer interactions and social rejection that traditional interventions work to address (Malecki & Elliott, 2002), suggesting that therapy dogs may provide additional support for these existing efforts.

The supporting specific students mechanisms revealed in this study demonstrate how therapy dogs can provide individualized social support that complements existing intervention strategies rather than replacing them. The community and belongingness effects suggest that, in addition to traditional approaches that emphasize individual skill building, therapy dogs may also support social integration, offering an additional pathway, which can work in conjunction with direct instruction approaches. Research on SEL emphasizes the importance of multiple, complementary approaches to developing social competence (Durlak et al., 2011; Zins et al., 2007). The empathy and compassion development documented through therapy dog interactions operates through natural modeling and emotional contagion in ways that may enhance structured SEL curricula, providing experiential learning opportunities that complement didactic instruction (Brackett et al., 2012).

The emotional well-being pathways shown in Figure 11 demonstrate traditional therapeutic models by demonstrating how therapy dogs provide support through presence and relationship that works alongside structured therapeutic activities. The joy and happiness manifestations documented in this study operate through spontaneous, authentic emotional responses that may create positive classroom climates conducive to learning, consistent with research on the importance of positive emotions in educational settings (Immordino-Yang & Damasio, 2007). The evidence that positive emotional states were consistently observable and created feedback loops that enhanced motivation suggests that relationship-based approaches may provide additional benefits when integrated with external reinforcement systems commonly used in Tier 3 settings (Lane et al., 2019).

The stress relief mechanisms documented in this research operate through naturally occurring interactions in ways that complement planned interventions, suggesting potential synergies between informal relational support and formal stress management instruction. The self-efficacy building documented in this study indicates that confidence may develop in students with histories of academic and social failure through multiple pathways. Traditional approaches emphasize structured success experiences and explicit skill instruction and remain essential components of Tier 3 interventions (Cook et al., 2008). The current findings suggest that students may also transfer confidence from therapy dog interactions to novel social situations, indicating that relationship-based approaches may provide additional benefits when integrated with task-based interventions for building self-efficacy in Tier 3 populations. This finding builds on self-efficacy theory emphasizing multiple sources of efficacy beliefs (Bandura, 1997).

The non-judgmental and trauma-informed care delivery illustrated in the model operates through unconditional acceptance and individualized responsiveness that aligns with trauma-informed educational practices (Branson et al., 2017). These findings suggest that relationship-based support may be accessible and effective for addressing trauma-related needs in educational settings when integrated with clinical approaches that emphasize formal treatment interventions. The evidence supports a collaborative approach integrating multiple forms of support for students with trauma histories in Tier 3 settings. The combination of these emotional well-being components creates a foundation that may enable the social and behavioral improvements documented across other domains.

The behavioral well-being outcomes displayed in Figure 11 provide evidence for examining self-regulation development and academic engagement in Tier 3 settings from multiple perspectives. The academic engagement effects illustrated in the model suggest that the relationship between emotional support and educational focus may be more complex than previously understood. Traditional views suggest that comfort provision may reduce academic motivation or distract from learning activities, and structured academic support remains crucial (Scott et al., 2019). However, the evidence that students maintained and enhanced academic engagement while accessing therapy dog support suggests that emotional safety may be a prerequisite for academic performance, indicating that AAIs and academic interventions may work synergistically rather than in competition. This finding aligns with neuroscience research on the relationship between emotional regulation and cognitive functioning (Immordino-Yang & Damasio, 2007).

The self-regulation acceleration documented in this research represents one of the most notable findings, with de-escalation time improvements from 45 min to one minute in some instances, suggesting that relationship-based approaches may provide efficient support when integrated with traditional behavioral interventions that rely on structured protocols and external management systems. This finding extends research on multi-component behavior support systems (Lane et al., 2019; Maggin et al., 2017), indicating that AAIs may enhance the efficiency of existing Tier 3 behavioral strategies. The motivation enhancement documented through therapy dog interactions operates through intrinsic mechanisms that may work alongside extrinsic mechanisms, offering an alternative perspective to traditional behaviorist reinforcement models (Skinner, 1953) that emphasize external contingencies, by showing how therapy dogs may foster intrinsic, relationship-based motivation, which complements token-based systems for maintaining engagement in Tier 3 populations. Research on self-determination theory supports the integration of both intrinsic and extrinsic motivation strategies (Ryan & Deci, 2000).

The responsibility development documented in this study provides insights into how accountability and ownership develop in students with behavioral difficulties. The evidence that students began taking social–emotional assessments more seriously and asking clarifying questions rather than completing forms perfunctorily suggests that therapy dogs may support intrinsic motivation for self-reflection that external accountability measures cannot reach. This finding aligns with research emphasizing the importance of student engagement in self-monitoring processes (Walker et al., 2004). The job satisfaction impacts on educators represent a critical finding that suggests a potential benefit of well-designed AAIs programs; rather than adding burden, they may reduce stress for educators in intensive intervention settings. The presence of the therapy dog as a co-educator in the classroom supports the classroom teacher and promotes positive student well-being (Harris & Binfet, 2022). The finding that therapy dogs provide additional intervention options while helping educators manage difficult situations more effectively suggests that well-designed AAIs may address workforce sustainability issues that threaten program effectiveness in intensive intervention settings. This finding extends research on teacher retention and well-being in challenging educational contexts (Dreer & Gouasé, 2021; Pas et al., 2012).

The cross-domain amplification effects shown in Figure 11 suggest the importance of examining intervention models that consider interactions across multiple domains rather than focusing on discrete outcomes within specific domains. The evidence that improvements in social well-being reliably catalyze behavioral and emotional gains suggests that intervention effectiveness may depend not only on targeting specific skill deficits through direct instruction, but also on creating synergistic effects across domains. This finding supports theoretical frameworks emphasizing interconnected development across social, emotional, and behavioral domains (Durlak et al., 2011; Zins et al., 2007).

The documented pathways between domains highlight the need to consider traditional evaluation approaches that examine single outcomes alongside systemic change patterns. The evidence that emotional regulation improvements enable enhanced academic engagement, which in turn facilitates peer relationship development, suggests that intervention impact may unfold through complex sequences that traditional pre-post assessment approaches may not fully capture. This finding aligns with research on dynamic systems approaches to understanding student development (Thelen & Smith, 1994).

This interconnected model invites the field to reconceptualize intervention intensity calculations. If improvements in one domain reliably produce gains in others, traditional effect size calculations may systematically underestimate AAIs impact by failing to account for cross-domain amplification effects. This finding has important implications for how the field evaluates complex, relationship-based interventions in educational settings and warrants further investigation using methodologies designed to capture multi-domain effects.

The Tier 3 context illustrated in Figure 11 reveals potential opportunities for AAIs implementation in intensive settings. The smaller class sizes, specialized staff training, and individualized support systems create conditions where therapy dogs can provide highly responsive, individualized support while systematic relationship building occurs across multiple domains simultaneously. These structural features align with recommendations for comprehensive intervention approaches in intensive settings (Freeman et al., 2006; Nitz et al., 2023). However, it is important to note that there are potential challenges to a Tier 3 classroom setting, such as noise, emotional dysregulation, and social conflict, which could potentially strain the therapy dog and create an unsafe environment. It is important for handlers to be aware of the signs that their therapy dog is stressed and to provide a safe space in the classroom, like the crate that was available for Muffin in the present study. We recommend that the frequency and duration of integration be closely monitored by the handler to ensure that the therapy animal has adequate rest days built in and that the overall number of days should be based on the individualized needs of the therapy animal, usually not to exceed three days per school week.

The safe learning environment principles embedded in the model align with CI3T research demonstrating that engaging educational contexts reduce dysregulated behavior (Lane et al., 2019). The evidence that therapy dogs enhance environmental safety while maintaining academic focus suggests that, when properly implemented, AAIs may help balance providing support and maintaining instructional priorities rather than creating tension between these goals. This finding suggests that well-implemented AAIs may support efforts to balance emotional support and academic achievement in ways that benefit both students and educators.

The evidence that therapy dogs can serve as relationship catalysts while providing successful interaction experiences indicates the potential value of relationship-based interventions alongside skill building approaches in Tier 3 settings. Both relationship repair and skill development appear important for addressing the complex social–emotional needs that bring students to Tier 3 settings. This finding is consistent with research emphasizing the importance of therapeutic relationships in educational interventions (Branson et al., 2017; Gueldner et al., 2020).

The case study approach employed in this research, building on methodological innovations demonstrated by Harris and Binfet (2022), reveals the sophisticated, dynamic processes that characterize effective AAIs implementation. The multi-modal analysis demonstrates that therapy dogs operate through contextual, relational mechanisms that may not be fully captured through standardized assessment approaches or experimental designs that prioritize control over ecological validity. This finding supports calls for methodological pluralism in intervention research (Cook et al., 2015).

The approach challenges traditional research paradigms in intervention science by demonstrating how complex interventions create effects through dynamic interactions between students, therapy dogs, educators, and environmental factors. The systematic documentation of moment-by-moment changes, relationship development patterns, and environmental modifications provides insights into intervention mechanisms that would be invisible through traditional quantitative approaches alone. These findings suggest the value of mixed-methods and qualitative approaches for understanding complex educational interventions (Stake, 1995).

The triangulation of video analysis, interviews, field notes, and member checking creates a comprehensive understanding of how therapy dogs influence classroom dynamics while maintaining scientific rigor. This approach addresses limitations in AAIs research that have relied heavily on self-report measures that may be inadequate for capturing the complex, relational nature of human–animal interactions in educational environments. The combination of methods used in this study provides a model for systematic examination of individual variation, environmental moderators, and implementation factors essential for understanding the well-being pathways illustrated in the figures while creating foundations for systematic replication across diverse Tier 3 settings.

### Limitations and Future Directions

While this research does reach in-depth insight about how a therapy dog intervention can be applied within a Tier 3 classroom to understand the impacts of the intervention on factors of mental health and well-being it does have some limitations. First, the findings of this study are not generalizable to all Tier 3 classroom settings, and the perspectives of the students and classroom teacher/handler are not representative of all teachers and students in classrooms with a therapy dog. Second, the number of participants in the study is small, with only one Tier 3 classroom participating in the research. While the district wide therapy dog program was large, there was only one therapy dog in a classroom at the school with the Tier 3 program.

Recommendations for future research are to explore more cases of Tier 3 programs with therapy dogs to obtain additional qualitative data from educators and students about their perspectives of the impacts of therapy dogs on mental health and well-being, as well as teaching and learning. We also recommend that observational data of the learning environment with and without the therapy dog present be recorded for longer periods of time, as well as times that focus on specific SEL support time. Future research should employ similar methodological approaches while also incorporating quantitative outcome measures to triangulate findings (Brelsford et al., 2017).

## 5. Conclusions

This case study demonstrates that therapy dog interventions can function as complimentary enhancements to existing Tier 3 interventions. Therapy dog interventions can be systematically integrated to build upon existing Tier 3 setting components to support integration at the student, staff, and school levels. This research demonstrates that therapy dogs can influence multiple domains for well-being, such as social, emotional, and behavioral, to address the social–emotional needs of students in Tier 3 settings. The evidence suggests that well-implemented AAIs can support educators with a therapy dog that is also a co-educator enhancing emotional support and academic achievement to benefit both students and educators.

## Figures and Tables

**Table 1:** Operational definitions of sub-themes.

Main Theme	Sub-Theme	Definition
Social Well-Being	Environment	Observations describing positive impacts on the physical and social learning environment.
	Peer Relationships	Interactions or social connections between students or between students and staff.
	Supporting Specific Students	Individualized support strategies or interventions targeting particular student needs.
	Community	Behaviors or statements indicating membership and integration within classroom or school groups.
	Belongingness	Expressions demonstrating sense of acceptance, inclusion, or being valued as a member.
	Empathy/Compassion	Manifestations of understanding, care, or emotional support toward others.
Behavioral Well-Being	Academic Engagement	Active participation, attention, or involvement in educational tasks and activities.
	Self-Regulation	Management of emotions, behaviors, or responses to maintain appropriate functioning.
	Motivation	Drive, interest, or willingness to participate in activities or pursue goals.
	Responsibility	Demonstration of accountability, thoughtful decision-making, or ownership of actions
	Job Satisfaction	Professional fulfillment, effectiveness, or positive attitudes toward work responsibilities.
Emotional Well-Being	Joy/Happiness	Observable expressions of positive emotions, contentment, or pleasure.
	Stress Relief	Reduction in tension, anxiety, or distress; increased comfort or relaxation.
	Self-Efficacy	Confidence in personal abilities to perform tasks or achieve desired outcomes.
	Non-Judgmental/Trauma Informed Care	Provision of unconditional acceptance and individualized support sensitive to past experiences.

## Data Availability

The datasets presented in this article are not readily available because of IRB requirements for participant confidentiality.

## Abbreviations

The following abbreviations are used in this manuscript:

AAI	Animal-assisted interventions
MTSS	Multi-tiered systems of support
SEL	Social emotional learning
